# Cu/Zn superoxide dismutase expression in the postnatal rat brain following an excitotoxic injury

**DOI:** 10.1186/1742-2094-2-12

**Published:** 2005-06-01

**Authors:** Hugo Peluffo, Laia Acarin, Maryam Faiz, Bernardo Castellano, Berta Gonzalez

**Affiliations:** 1 Unit of Histology, Department Of Cell Biology, Physiology, and Immunology; Autonomous University of Barcelona, 08193, Spain; 2 Institute of Neuroscience, Autonomous University of Barcelona, 08193, Spain

## Abstract

**Background:**

In the nervous system, as in other organs, Cu/Zn superoxide dismutase (Cu/Zn SOD) is a key antioxidant enzyme involved in superoxide detoxification in normal cellular metabolism and after cell injury. Although it has been suggested that immature brain has a different susceptibility to oxidative damage than adult brain, the distribution and cell-specific expression of this enzyme in immature brain and after postnatal brain damage has not been documented.

**Methods:**

In this study, we used immunohistochemistry and western blot to analyze the expression of Cu/Zn SOD in intact immature rat brain and in immature rat brain after an NMDA-induced excitotoxic cortical injury performed at postnatal day 9. Double immunofluorescence labelling was used to identify Cu/Zn SOD-expressing cell populations.

**Results:**

In intact immature brain, Cu/Zn SOD enzyme was widely expressed at high levels in neurons mainly located in cortical layers II, III and V, in the sub-plate, in the pyriform cortex, in the hippocampus, and in the hypothalamus. Glial fibrillary acidic protein-positive cells only showed Cu/Zn SOD expression in the glia limitans and in scattered cells of the ventricle walls. No expression was detected in interfascicular oligodendroglia, microglia or endothelial cells. Following excitotoxic damage, neuronal Cu/Zn SOD was rapidly downregulated (over 2–4 hours) at the injection site before neurodegeneration signals and TUNEL staining were observed. Later, from 1 day post-lesion onward, an upregulation of Cu/Zn SOD was found due to increased expression in astroglia. A further increase was observed at 3, 5 and 7 days that corresponded to extensive induction of Cu/Zn SOD in highly reactive astrocytes and in the astroglial scar.

**Conclusion:**

We show here that, in the intact immature brain, the expression of Cu/Zn SOD was mainly found in neurons. When damage occurs, a strong and very rapid downregulation of this enzyme precedes neuronal degeneration, and is followed by an upregulation of Cu/Zn SOD in astroglial cells.

## Background

It has been shown that ~2–5% of the electron flow in isolated brain mitochondria produces superoxide anion radicals (O_2_^-.^) and hydrogen peroxide (H_2_O_2_) [1]. Cellular levels of O_2_^-. ^are normally low due to the action of cytosolic copper zinc superoxide dismutase (Cu/Zn SOD) and mitochondrial manganese superoxide dismutase (Mn SOD). These key enzymes catalyze the dismutation of O_2_^-. ^to oxygen and H_2_O_2 _[2]. Increased production of superoxide and its derivatives can induce injury by diverse mechanisms including initiation of lipid peroxidation, inactivation of enzymes, damage to DNA, and protein sulfhydryl oxidation. In particular, in the presence of nitric oxide (NO^.^), O_2_^-. ^and NO^. ^rapidly and spontaneously react to form the potent oxidant peroxynitrite (ONOO^-^), which is capable of nitrating tyrosine [3,4] contributing to the neuropathological process [5]. In this sense, superoxide radicals have been identified as important mediators of oxidative injury during ischemia-reperfusion and many other neurological injuries [6]. Cerebral ischemia [7-9] and traumatic brain injury [10] cause a rapid and sustained increase in the formation of O_2_^-.^, which is accelerated during mitochondrial dysfunction, and may also result from increased activity of several cytosolic enzymes as phospholipase A_2 _[11] or cycloxygenase 2 (COX2) [7].

In the adult central nervous system (CNS), Cu/Zn SOD is widely expressed in different neuronal populations: hippocampal CA pyramidal neurons and granule neurons of the dentate gyrus, cortical neurons, especially pyramidal cells, neurons of the substantia nigra, and at very high levels in motor neurons of the spinal cord [12-17]. In regards to glial cells, it is generally assumed that microglial cells and oligodendrocytes do not show expression of Cu/Zn SOD [12,16-19], but the astroglial expression of this enzyme is controversial. Some studies have found Cu/Zn SOD expression primarily and most intensely in astrocytes [18,19], but a number of works have failed to detect this expression in astrocytes [13-15,17], or have reported expression only in some scattered astrocytes [12,16]. On the other hand, expression of Cu/Zn SOD has been well documented in reactive astrocytes several days after different types of adult brain injury, like transient cerebral ischemia [13], kainate treatment [14], quinolinic treatment [18], or Alzheimer's and Down's Syndrome [16].

According with its antioxidant role, in most adult CNS injury models the over-expression of Cu/Zn SOD is thought to be neuroprotective [20-22]. In agreement, synthetic O_2_^-. ^dismuting metalloporphyrins protect against transient middle cerebral artery occlusion [23], and targeted deletion of the Cu/Zn SOD or extracellular SOD genes worsens outcome after focal ischemia [24,25]. However, contradictory results have been reported regarding the toxicity of O_2_^-. ^after hypoxic/ischemic injury to the immature brain. Whereas several antioxidant molecules including SOD mimetics (as O_2_^-. ^dismuting metalloporphyrins) have been shown to be neuroprotective [26], a slightly worsened neuropathological outcome is observed in transgenic mice over-expressing Cu/Zn SOD [27]. Several other differences in regards to oxidative stress and antioxidant defences have been reported in the immature versus the mature brain. For example, and in comparison to the adult brain, in the immature damaged brain glutathione peroxidase is not upregulated after trauma [28]; free iron accumulates more rapidly within 4 hours after transient cerebral ischemia stimulating Fenton reactions [29,30]; and metallothioneins, potent antioxidant enzymes that bind transition metals as Zn, are less concentrated [31,32]. Finally, the postnatal brain is more sensitive than the adult brain to the neurotoxic actions of N-methyl-D-aspartate (NMDA) [33], which will lead to increased O_2_^-. ^generation [34,35]. Interestingly, it has been reported that Cu/Zn SOD is rapidly downregulated after several types of injury in the mature CNS [13,14,36,37], but no data is available regarding this phenomenon in immature brain, where overall findings suggest a differential scenario for oxygen and nitrogen reactive species in the evolution of a brain injury [38].

In this context, the aim of our study was to evaluate the temporal and spatial dynamics, and the identity of Cu/Zn SOD expressing-cells, in the intact immature rat brain, and following an excitotoxic injury.

## Methods

### Excitotoxic lesions

Nine-day-old Long-Evans black-hooded rat pups of both sexes were placed in a stereotaxic frame adapted for newborns (Kopf) under isofluorane anaesthesia. The skull was opened using a surgical blade, and 0.15 μl of saline solution (0.9% NaCl, pH 7.4) containing 18,5 nmol of N-methyl-D-aspartate (NMDA) (Sigma, M-3262, Germany) were injected into the right sensorimotor cortex at the level of the coronal suture (2 mm lateral from bregma and at 0.5 mm of depth). Control animals received an injection of 0.15 μl of vehicle saline solution. After suture, pups were placed in a thermal pad and maintained at normothermia for 2 hours before being returned to their mothers. Experimental animal work was conducted according to Spanish regulations, in agreement with European Union directives. This experimental procedure was approved by the ethical commission of the Autonomous University of Barcelona. All efforts were made to minimize animal suffering in every step.

### Immunocytochemical study

Intact immature brains from P9, P10, P12, and P16 rats of both sexes where used for the analysis of Cu/Zn SOD expression under physiological conditions (2–4 animals per time). Moreover, at 2, 4, and 10 hours, and 1, 3, 5 and 7 days after NMDA (3–4 animals per time) or saline injection (2 animals per time), rats were anaesthetized and perfused intracardially with 4% paraformaldehyde in 0.1 M phosphate buffer (pH 7.4). Brains were post-fixed in the same fixative for 2 hours and sunk in a 30% sucrose solution before being frozen with dry CO_2_. Coronal sections (30-μm-thick) were obtained using a Leitz cryostat. Free-floating parallel sections were treated with 10% foetal calf serum in Tris-buffered saline (TBS: 0.05 M Trizma base containing 150 mM of NaCl, pH 7.4) +1% triton X-100 for 1 hour, and incubated overnight at 4°C with sheep polyclonal anti-Cu/Zn SOD (574597, Calbiochem, Darmstad, Germany) (1:300) in the same solution. Afterwards, sections were rinsed and incubated for 1 hour at room temperature with Cy3-conjugated anti-sheep secondary antibody (AP147C, Chemicon, California, USA) (1:150). As negative controls, sections were incubated in media lacking the primary antibody.

### Double labelling with specific cell markers

Double labelling was carried out in order to identify Cu/Zn SOD-expressing cells in sections previously immunoreacted for Cu/Zn SOD as reported above.

For neuronal identification, sections were further incubated with a monoclonal anti-NeuN antibody (MAB377, Chemicon, California, USA) (1:1000) and Cy2-conjugated anti-mouse secondary antibody (PA-42002, Amersham Pharmacia Biotech, England) (1:1000).

For astroglial labelling, sections were incubated with a polyclonal anti-GFAP antibody (Z-0334, Dakopatts, Denmark) (1:1800), and immunostaining was visualized with Cy2-conjugated anti-rabbit secondary antibody (PA-42004, Amersham Pharmacia Biotech, England) (1:1000).

As a microglial marker, sections were processed for double staining with tomato lectin histochemistry by incubating with the biotinylated lectin from Lycopersicon esculentum (tomato) (Sigma, L-9389, Germany) diluted to 6 μg/ml, followed by Cy2-conjugated streptavidin (PA-42000, Amersham Pharmacia Biotech, England) (1:1000).

Selected sections of all double labelling techniques were incubated for 5 min with a 0.00125 μg/ml solution of 4, 6-diamino-2-phenylindole (DAPI) in TBS. Double-stained sections were analyzed using a Nikon Eclipse E600 epifluorescence microscope and a LEICA TCS SP2 AOBS confocal microscope.

### TUNEL labelling

Terminal dUTP Nick End Labelling (TUNEL) staining for detection of DNA fragmentation was performed on parallel sections mounted on slides. Tissue sections were rinsed in Tris buffer (10 mM, pH 8) and EDTA (5 mM) and then incubated in the same buffer plus Proteinase K (20 μg/ml) for 15 min. at room temperature. After several washes with EDTA (5 mM), sections were incubated for 10 min. in TdT buffer (Tris 30 mM, 140 mM Sodium Cacodilate, 1 mM Cobalt chloride, pH 7.7). Sections were then incubated in TdT buffer plus 0.161 Units/ μl TdT enzyme (Terminal Transferase, 3333566 Roche, Manheim, Germany) and 0.0161 nmol/ μl of biotin-16-dUTP (1093070, Roche, Manheim, Germany) for 30 min. at 37°C. The reaction was stopped by washing the sections in citrate buffer (300 mM sodium chloride, 30 mM sodium citrate, 5 mM EDTA). After several washes with TBS, sections were incubated with avidin-peroxidase (P-0364, Dakopatts, Denmark) (1:400) for another hour at room temperature. Finally, the peroxidase reaction product was visualised in 100 ml of Tris buffer containing 50 mg of 3'-diaminobenzidine and 0.01% of hydrogen peroxide.

### Western blotting and densitometry

Three or four NMDA-injected and two intact and saline-injected animals for each survival time were decapitated, and the complete injected cortex quickly extracted, chopped and frozen in liquid nitrogen. Samples were resuspended in Tris/HCl (50 mM, pH7.8), EDTA (1 mM), DTT (1 mM), PMSF (100 μg/ml), pepstatin A (2 μg/ml), leupeptin (2 μg/ml), trypsin inhibitor (10 μg/ml), benzamidine (0.2 mM) and submitted to mechanical dissociation. Total protein concentration was measured by the bicinchoninic acid method and equal quantities of protein were run on a 15% SDS-polyacrilamide gel electrophoresis (SDS-PAGE). Electrotransfered protein samples to polyvinylidene fluoride (PVDF) membranes were incubated overnight at 4°C with TBS+0.3% Tween 20 and 5% non-fat milk, and for 2 hours at room temperature with sheep polyclonal anti-Cu/Zn SOD (574597, Calbiochem, Darmstad, Germany)(1:1000). Membranes were rinsed and incubated in biotinylated anti-sheep antibody (1:1000) (RPN-1025, Amersham Pharmacia Biotech, England), avidin-peroxidase (1:2000) (P0364, Dakopatts, Denmark) and finally in the chemiluminiscent substrate SuperSignal West Pico (PIERCE) combined with exposure on Hyperfilm ECL (Amersham).

Semi-quantitative estimation of western blot protein signals were performed by measuring band integral intensity with analySIS^® ^software after high resolution scanning.

### Statistical analysis

All results are expressed as mean ± standard error mean (SEM). ANOVA followed by Fisher's PLSD post-hoc test was used to determine significant differences (p<0.05) after western blot densitometry.

## Results

### Cu/Zn superoxide dismutase expression in the immature rat brain

**Figure 1:**
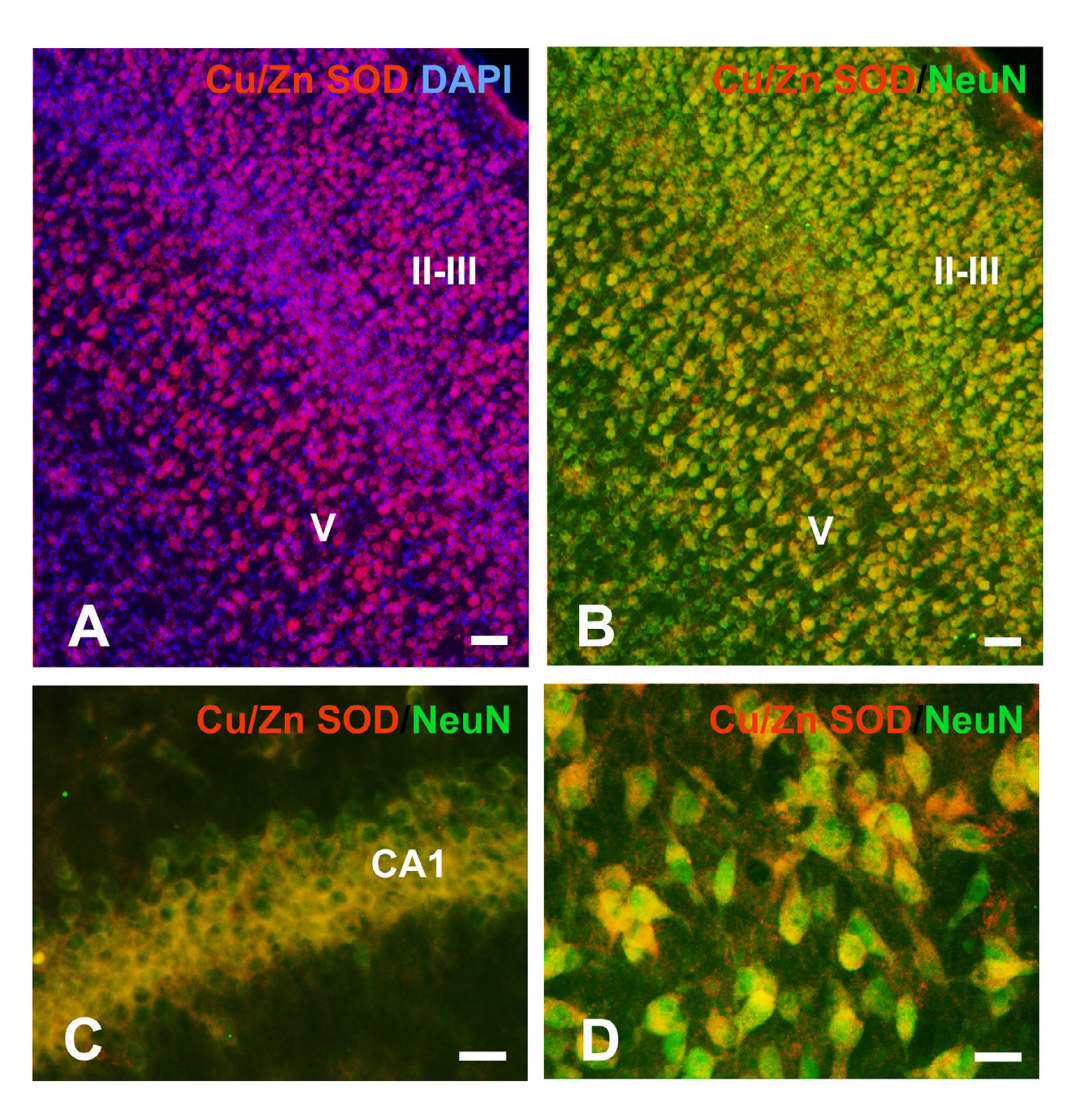
**Neuronal Cu/Zn SOD immunoreactivity in intact immature brain. **Widespread immunoreactivity for Cu/Zn SOD was observed mainly in neurons, both in the cytoplasm and nucleus, sparing the nucleolus. In the cortex, the highest expression was observed in cortical pyramidal neurons of layers V and III and II (**A**, **B**: Parietal cortex). In addition, the hippocampal pyramidal layer (CA1; **C**), and of substantia nigra (**D**) also displayed immunoreactivity. Scale bars: 50 μm in A and B; 20 μm in C and D.

**Figure 4:**
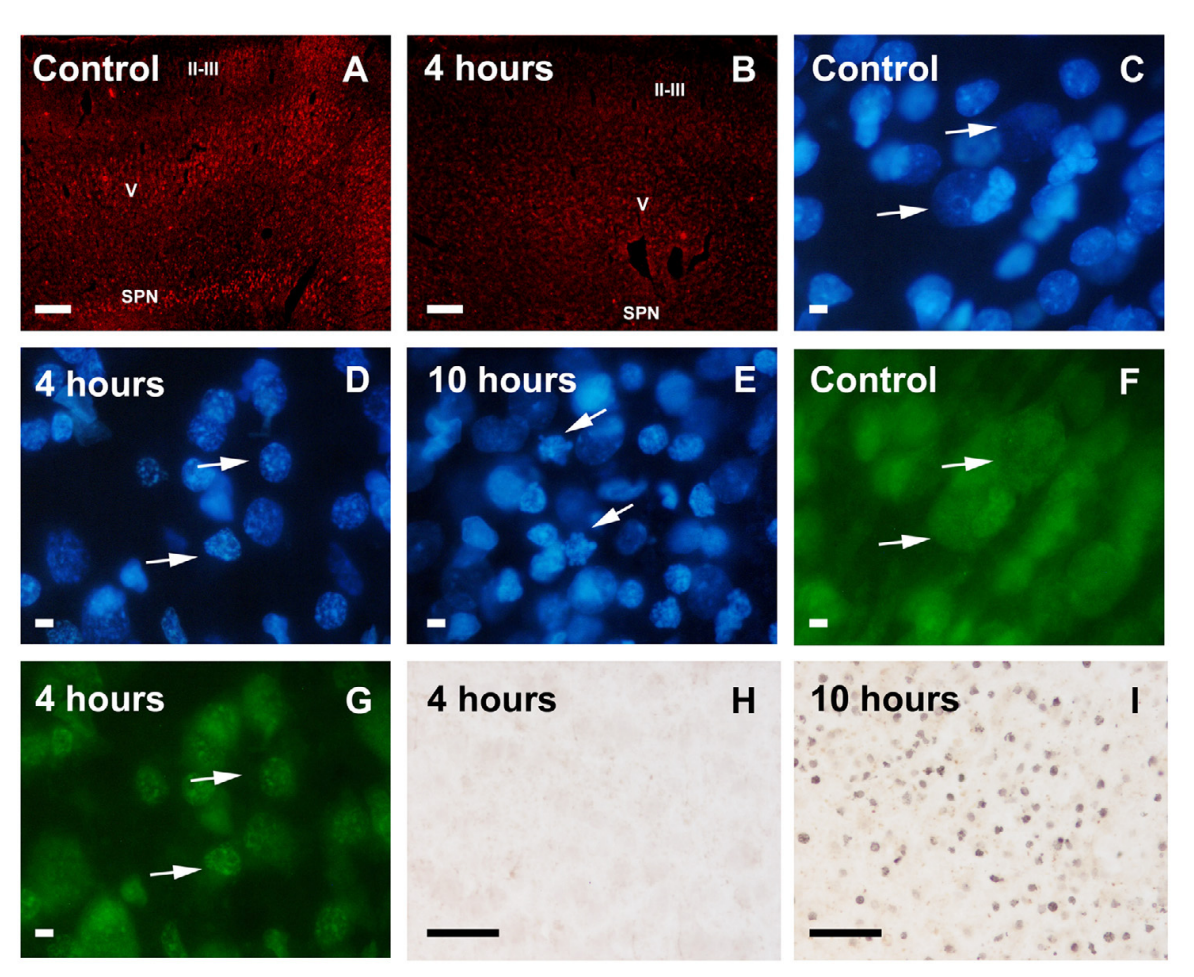
**Cell-specific Cu/Zn SOD expression in the early excitotoxicaly lesioned immature brain. **In the injection site, Cu/Zn SOD immunoreactivity decreased from 2–4 hours after the NMDA injection, and continued to be downregulated until 24 hours after injection (**A-B**,**SPN**: sub-plate neurons, **V **and **II-III**: cortical layers). Neurons from the injected cortex after 4 hours showed slightly condensed chromatin (compare **C **and **D**: arrows), and a shift of NeuN staining from the soma and nucleus towards the nucleus (compare **F **and **G**: arrows) in the absence of TUNEL staining (**H**). From 10 hours after lesion onward, neuronal nuclei showed clear apoptotic signals like condensation and blebbing (**E**: arrows) and TUNEL staining (**I**). Scale bars in A-B: 200 μm; in C-G 5 μm; and in I-H: 40 μm.

As there is no available description of Cu/Zn SOD cell-specific expression in the immature brain, we began our study with this general description. No changes in the distribution pattern and cellular identity of Cu/Zn-expressing cells were found between P9 to P16 and thus will be described in general. The immature rat brain showed widespread immunoreactivity for Cu/Zn SOD, mainly located in neurons. The most intense staining was observed in pyramidal neurons located in cortical layers V, III and II (Fig. 1A, B), in the pyriform cortex, and in the pyramidal neurons of the olfactory tubercle. Sub-plate neurons, a transient cell population located beneath the cortical plate [39] were also among the most intense cells (Fig 4A). In addition, hippocampal CA pyramidal neurons and some inter neurons (Fig. 1C), as well as dentate gyrus granular and sub-granular neurons, medial septum neurons, and hypothalamic neurons also displayed intense staining. There was also more diffuse staining in many other neuronal populations as for example in various thalamic nuclei, substantia nigra (Fig. 1D), and striatum. Most of the immunoreactivity was mainly observed in neuronal soma although less intense staining was also present in the nuclei, sparing the nucleolus.

**Figure 2:**
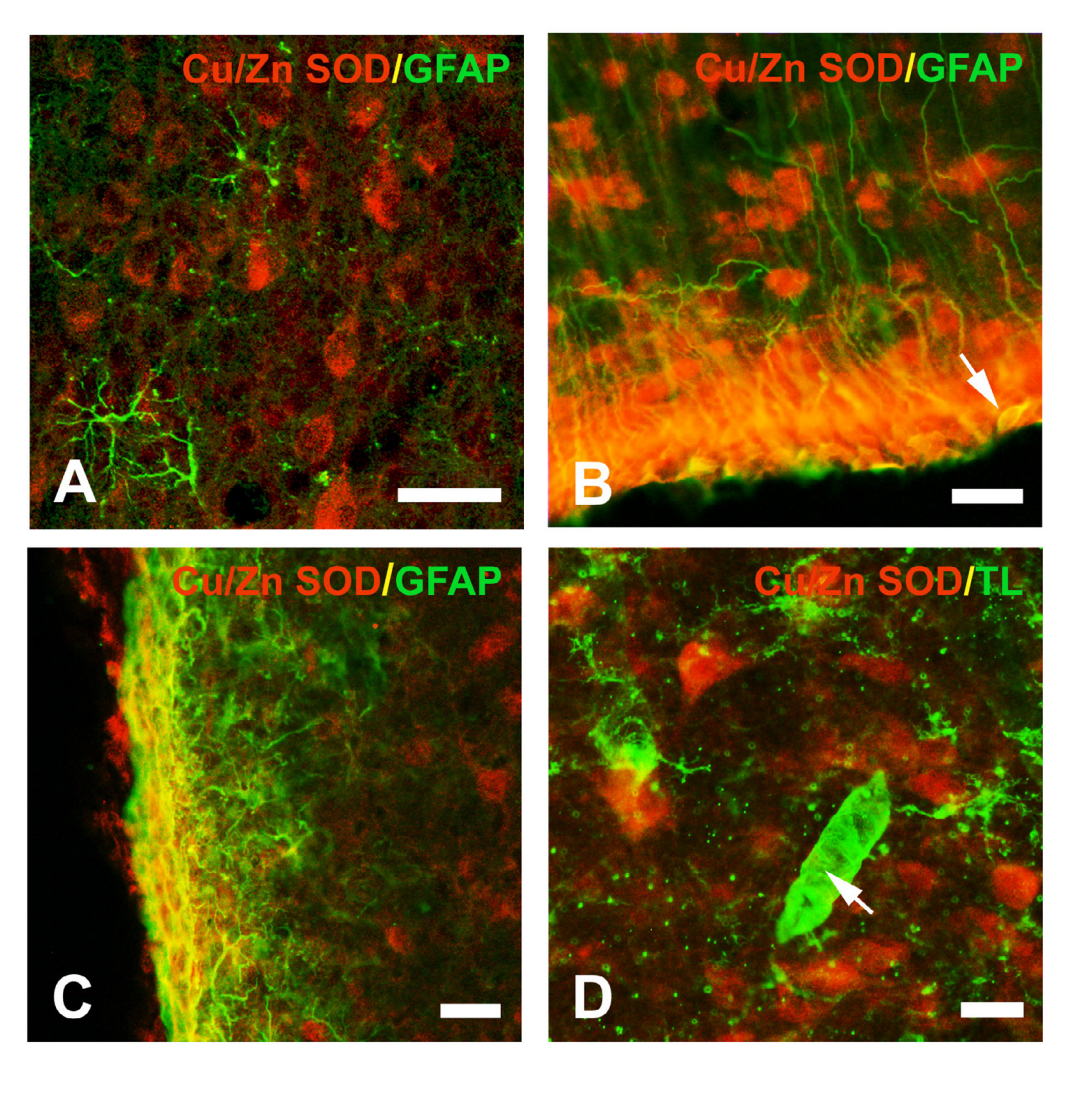
**Glial distribution of Cu/Zn SOD immunoreactivity in intact immature brain. **Cu/Zn SOD was not observed in the GFAP-expressing astrocyte population of the brain parenchyma (**A**: cortex, confocal image). However, GFAP-expressing astrocytes showed co-localization with Cu/Zn SOD in the glia limitans (**C, **confocal image) and in the ventricle walls (**B**: third ventricle; arrow: tanycyte). Microglial cells and endothelium (arrow), identified with tomato lectin histochemistry, were negative for Cu/Zn SOD (**D**: cortex; confocal image). Scale bars in A: 40 μm; in B, C and D: 20 μm.

No specific Cu/Zn SOD immunoreactivity was detected in parenchymal grey matter GFAP-positive astrocytes (Fig 2A). However, co-expression of Cu/Zn SOD and GFAP was observed in scattered tanycytes of the third ventricle wall (Fig 2B), in *corpus callosum*, and in astrocytes forming the glia limitans (Fig 2C). Most ciliated ependymal cells expressed Cu/Zn SOD (Fig. 2B). Moreover, besides the ependymal cells described above, co-localization confocal studies with tomato lectin, which specifically detects microglial cells as well as endothelium [40], did not show overlap with Cu/Zn SOD staining (Fig 2D). No consistently Cu/Zn SOD-labelled cells were observed in white matter tracts, which indicated that oligodendrocytes at this location do not seem to express this enzyme.

**Figure 3:**
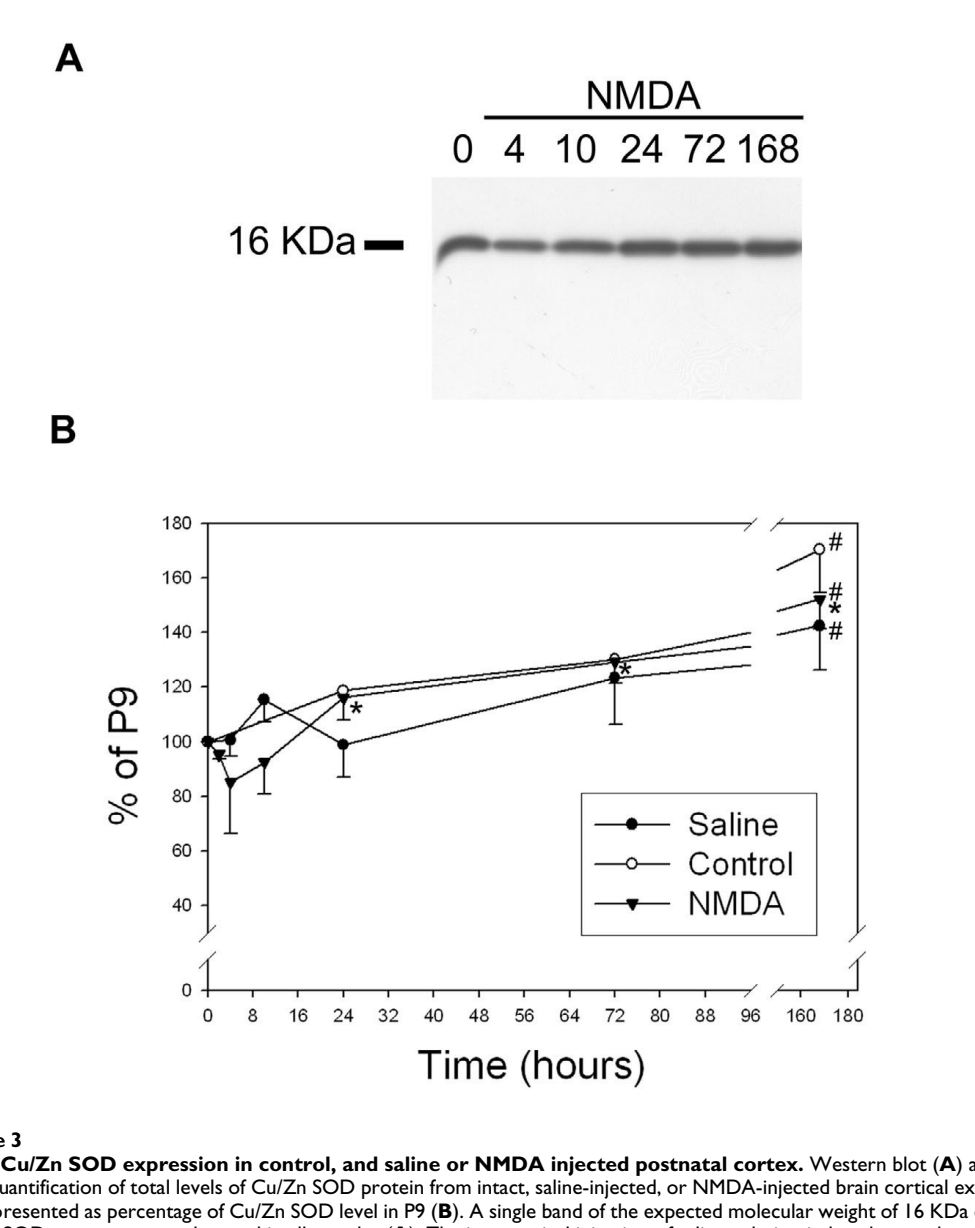
**Total Cu/Zn SOD expression in control, and saline or NMDA injected postnatal cortex. **Western blot (**A**) and semi-quantification of total levels of Cu/Zn SOD protein from intact, saline-injected, or NMDA-injected brain cortical extracts are represented as percentage of Cu/Zn SOD level in P9 (**B**). A single band of the expected molecular weight of 16 KDa of the Cu/Zn SOD monomer was observed in all samples (**A**). The intracortical injection of saline solution induced a trend towards the transient upregulation of Cu/Zn SOD which showed a peak at 10 hours post-injection (**B**: solid circles). In contrast, after NMDA injection, total cortical Cu/Zn SOD expression showed a trend towards rapidly diminishing (**A**, **B**: triangles). This trend of Cu/Zn SOD reduction at 4 hours after NMDA injection in total cortical extracts is comparable to the % of lesioned cortex which was around 20% (not shown). However, at later time points, Cu/Zn SOD was significantly upregulated peaking at 7 days (168 hours) post-lesion. In intact control brain (**B**: opened circles), total enzyme level significantly increased from postnatal day 9 to postnatal day 16. (# p < 0.05 in relation to P9 control animals and * p < 0.05 in relation to NMDA 4 hours).

Finally, western blots showed a significant developmental increase in the total amount of brain Cu/Zn SOD between P9 and P16 (Fig. 3B), evidenced as a single band of 16 KDa corresponding to the expected molecular mass of the enzyme monomer.

Control intracortical saline injection caused a local and transient increase in Cu/Zn SOD immunoreactivity mainly in cortical neurons (data not shown) 10 hours later surrounding the injection site. A trend towards a rapid and transient increase could also be observed by western blots (Fig 3B).

### Cu/Zn superoxide dismutase expression after excitotoxic injury to the immature brain

As shown before, intracortical NMDA administration is a model of excitotoxic damage that triggers rapid neuronal death and tissue injury, which expands rostro-caudally and includes part of the cortex, *corpus callosum*, dorsal striatum, septum and rostral hippocampus [33,41].

**Figure 5:**
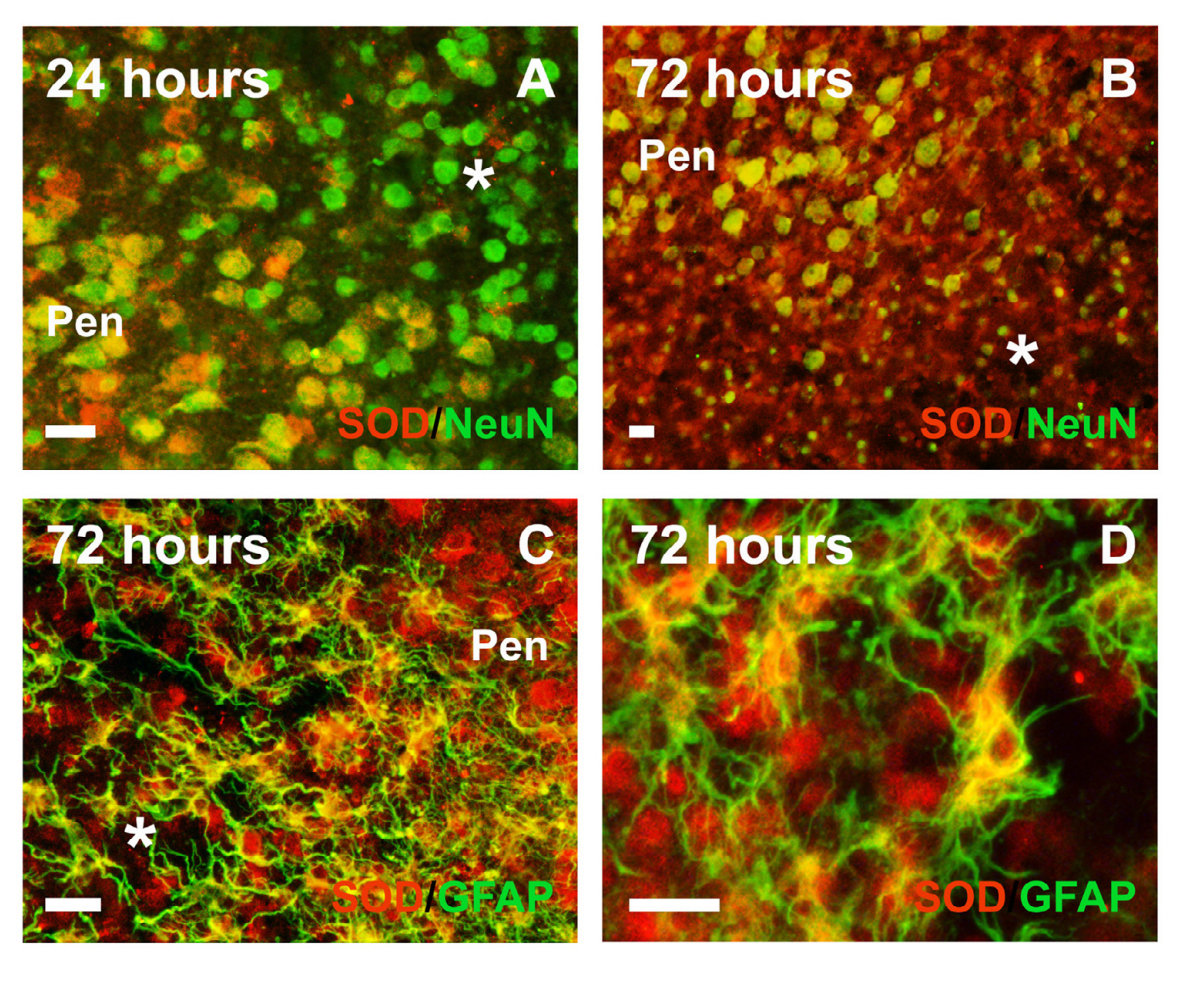
**Cell-specific Cu/Zn SOD expression in the late excitotoxicaly lesioned immature brain. **One day after the lesion (**A**), neuronal bodies were condensed in the lesioned zone (*) when compared to the penumbra zone (**Pen**). Moreover, the lesion core (*) showed very low or no expression of Cu/Zn SOD. In contrast, 3 days after injection, cortical expression of Cu/Zn SOD increased in the whole lesion (**B**, *). Neurons in the penumbra (**B**, **Pen**) and reactive hypertrophic astrocytes (**C**) within the degenerative core (*) expressed Cu/Zn SOD after 3 days. These GFAP-positive hypertrophic astrocytes were the main cell type expressing Cu/Zn SOD in the lesion core (**D**). This pattern of expression was maintained until 7 days post-lesion (the last time analyzed), when the glial scar was evident. Scale bars in A-D: 20 μm.

In contrast to saline injected animals, as observed both by western blot tendencies (Fig. 3) and immunohistochemistry (Fig. 4, 5) at 2–4 hours after NMDA injection, the lesioned neural parenchyma showed a drastic downregulation of total Cu/Zn SOD immunoreactivity in neuronal cells, which returned to basal levels 24 hours after the lesion and increased significantly thereafter due to expression in astroglial cells. Interestingly, the early decrease in neuronal Cu/Zn SOD immunoreactivity at 2–4 hours (Fig. 4A–B) was accompanied by a slight condensation of nuclear chromatin (Fig. 4C–D) and changes in NeuN neuronal marker (Fig. 4F–G), in the absence of apparent signs of neuronal degeneration or TUNEL staining (Fig. 4H). At later time points, from 10 hours, degenerating neurons still showed downregulated Cu/Zn SOD immunoreactivity, but displayed condensed nuclei, nuclear blebbing (Fig. 4E) and also DNA fragmentation observed by TUNEL staining (Fig. 4I). Downregulation of Cu/Zn SOD and signs of neuronal degeneration were observed from 10 hours in primary cortical degenerating areas and from 1 day post-lesion in secondary degenerating regions such as the cortical, striatal and hippocampal penumbra (Fig. 5A). However, the total Cu/Zn SOD enzyme level increased from 1 day post-lesion, reaching a maximum induction after 3–7 days (Fig 3B and 5B), due to astroglial upregulation of the enzyme. Cu/Zn SOD was induced in the soma and proximal projections of the most hypertrophied and most intensely GFAP-immunopositive astroglial cells of the whole degenerative area including the white matter (Fig. 5C), but not in slightly activated astrocytes with lower GFAP immunoreactivity. This elevated expression continued at 5 and 7 days post-lesion when most astrocytes of the degenerative area were strongly hypertrophic and formed the glial scar (Fig. 5D). Only scattered reactive microglial cells expressed Cu/Zn SOD at 3 days after lesion, but most reactive microglial cells of the lesioned parenchyma remained negative.

## Discussion

In the last decade, progress has been made regarding the complex evolution of damage in the developing brain [38] and how it differs with adult brain injury. In this sense, one of the divergences proposed is that immature brain deals poorly with oxidative stress. In this study, we have shown that the expression of the key antioxidant enzyme Cu/Zn SOD is mainly found in neuronal cells. When damage occurs, a strong neuronal downregulation of this enzyme precedes both neuronal cell death and the subsequent Cu/Zn SOD upregulation in astroglial cells.

### Cu/Zn SOD in the intact immature brain

It is well known that total brain levels of antioxidant enzymes vary throughout life [32,41,42]. Accordingly, brain glutathione peroxidase and Mn SOD were reported to increase during the first month of postnatal rat life, and to continue increasing slightly during adulthood and aging [28,43-46]. Catalase has been reported to peak around the first week of life and then decline to reach a plateau by the first month [43,44]. Regarding Cu/Zn SOD, it is known that its levels increase rapidly after birth, peaking around the second postnatal week, in agreement with our western blot studies. Later on, Cu/Zn SOD decreases slightly to reach adult levels, but increases slightly again in aging [42,44,45]. Thus, it seems clear that immature rat brain has a different balance of antioxidant enzymes, which reach the adult overall pattern only after the first month of life.

We have shown that in the postnatal brain the majority of Cu/Zn SOD immunoreactivity is observed in neuronal somas with less intense staining present in the nuclei, sparing the nucleoli, as has been reported earlier for adult animals [12-17,47]. Accordingly, even though changes in Cu/Zn SOD levels occur in postnatal development, the cell type distribution of this enzyme does not change. Neurons are the brain cells with the highest oxygen consumption, constantly submitted to oxidative stress as it is suggested by O_2_^-. ^mediated oxidation of hydroethidine [9], or by the presence of nitrotyrosine [48,49]. Accordingly, it is not surprising that high levels of Cu/Zn SOD are observed in a wide number of neuronal populations, specifically in the large neurons with high energetic requirements, like pyramidal neurons, or catecholaminergic neurons which are submitted to elevated oxidative stress due to catecholamine metabolism [50]. Very recently, a site for the pro-inflammatory transcription factor NFκB has been reported at the human Cu/Zn SOD promoter, which can induce its expression [51]. Accordingly, in the CNS, neurons are the main cells that display constitutively activated NFκB [52,53], and its activity is required for their survival [54]. In addition, the other important cellular superoxide dismutase, the Mn SOD, is also enriched in subsets of neurons [55], although different from those enriched in Cu/Zn SOD.

No immunoreactivity for Cu/Zn SOD was observed in the GFAP-expressing astrocyte population of the neural parenchyma in the immature brain, as has been previously reported for adult brain [13-15,17,47], and despite the fact that immature astrocytes differ from adult astrocytes. This lack of Cu/Zn SOD expression contrasts with the higher tolerance of astrocytes to oxidative stress [48,56]. Accordingly, and in comparison with neuronal cells, astrocytes in culture have been shown to have higher levels of glutathione and the lipophilic antioxidant vitamin E [57], and they are capable of synthesizing their own glutathione from cysteine [58], mechanisms that probably confer upon astrocytes an elevated antioxidant status and increased resistance towards oxidative stress, despite the absence of detectable levels of Cu/Zn SOD. Noteworthy, Cu/Zn SOD immunoreactivity is only found in glia limitans astrocytes and in GFAP-positive tanycytes, which could be attributed to the contact of these astrocytes with non-CNS molecules, which are also known to induce the expression of several other activation markers observed previously in these regions, like GFAP overexpression, vimentin or metallothioneins [41]. Regarding microglial cells, neither resting ramified microglia nor amoeboid microglial cells found in the immature brain showed Cu/Zn SOD immunoreactivity, as has been shown for adult resting microglial cells [12,17,47]. In addition, we were unable to find consistent immunoreactivity for Cu/Zn SOD in white matter oligodendrocytes of immature brain, as has been described in the adult [12,17,47]. The lack of this enzyme could help explain the well known high sensitivity of oligodendrocytes to excitotoxicity and oxidative stress [59], especially in the immature brain where white matter injury is thought to be the underlying mechanism of brain damage [38].

### Cu/Zn SOD in the injured immature brain

After an excitotoxic injury to the postnatal brain, we have observed a dramatic and rapid neuronal downregulation of Cu/Zn SOD in the NMDA injection site, which is early evident 2–4 hours after injection in neurons that only show slight and very early signs of degeneration and are negative for TUNEL staining. In fact we have previously shown that these neurons display, 10 hours after NMDA injection, NFκB activation and COX2 upregulation, suggesting that they are still active and functional [53,60]. The Cu/Zn SOD downregulation also coincides with neuronal nitration [48] suggesting endogenous O_2_^-.^/peroxynitrite formation at these very early time points in compromised neurons. Although to our knowledge this is the first study describing the expression of Cu/Zn SOD after immature brain damage, studies on adult brain injury have also shown, as early as 4 hours after transient cerebral ischemia, Cu/Zn SOD immunoreactivity downregulation in striatum and cortex [37] and in neurons of the hippocampal CA region [13]. Moreover, 24 hours after injection of kainate to adult rat hippocampus, Cu/Zn SOD immunoreactivity is also downregulated in neurons of CA, despite an absence of apparent neuronal degeneration at that time-point [14]. Accordingly, it is known that superoxide scavengers protect from injury at these early time points [23,26,61]. Although the mechanism whereby this rapid downregulation occurs is not clear, it appears that it could be mediated by oxidative stress, as PC12 cells treated with H_2_O_2 _rapidly (after 4 hours) downregulate Cu/Zn SOD [51]. Interestingly, hypothermia, the most powerful neuroprotective strategy known, not only inhibits the rapid downregulation of Cu/Zn SOD after a traumatic brain injury but also induces its over-expression [36]. Most surprisingly, this effect is specific for Cu/Zn SOD, and in fact hypothermia induces a less significant upregulation of other antioxidant enzymes such as catalase and glutathione peroxidase in comparison with non-hypothermic brain.

Contradictory results have been reported regarding the toxicity of O_2_^-. ^after hypoxic/ischemic injury to the immature brain. Whereas slightly worsened neuropathological outcome was observed in transgenic mice overexpressing Cu/Zn SOD and submitted to severe hypoxia/ischemia [27], several antioxidant molecules including SOD mimetics as O_2_^-. ^dismuting metalloporphyrins were shown to be neuroprotective [26]. One hypothesis is that Cu/Zn SOD transgenic mice produce excess H_2_O_2 _that in the immature brain is not cleared by the upregulation of the glutathione peroxidase as has been reported for adult animals [28,62]. However, an additional explanation would be that as the postnatal brain express increased amounts of the NMDA receptor [63], whose activation leads to increased O_2_^-. ^generation [34,35], an initial enhanced oxidative stress would occur. Taken together, this data suggest that in the immature brain subjected to ischemia/excitotoxicity, neurons at the lesion zone are very early submitted to an elevated oxidative stress, and therefore Cu/Zn SOD downregulation contributes to the further amplification of cell damage and neuronal cell death.

Although Cu/Zn SOD expression remains very low in compromised neurons, a return to normal expression levels is seen by 24 hours after lesion, and an increase in total enzyme level is observed later on. This secondary Cu/Zn SOD induction is due to upregulation in reactive hypertrophic astrocytes within the lesion site. We have shown in previous studies that these reactive astrocytes display activated NFκB from 10 hours after the excitotoxic lesion [53], which could contribute to the induction of the Cu/Zn SOD observed here. We have also shown that the hypertrophic Cu/Zn SOD-overexpressing astrocytes are heavily nitrated, and also display metallothionein I-II expression, suggesting an elevated rate of oxidative stress in this particular group of cells [48]. Our findings in immature brain are in accordance with studies of adult brain damage that have shown induction of Cu/Zn SOD and Mn SOD in astrocytes several days after focal ischemia [37] excitotoxicity [14,18], or in Alzheimer's disease and Down's Syndrome [16]. In addition to the upregulated enzymatic antioxidant defences, astrocytes have been shown to produce their own glutathione and also provide neurons with cysteine, a rate-limiting precursor in neuronal glutathione synthesis [58]. Thus, astrocytes seem to be the main cell type increasing the total antioxidant capabilities in the nervous tissue after a lesion, which can in addition explain their elevated resistance to cell death after an injury. In this sense, previous studies showed that Cu/Zn SOD-overexpressing astrocytes have increased resistance to oxidative damage [64] and attenuated oxidative inhibition of glutamate uptake [65], allowing for a maintenance of their physiological functions after a lesion.

Regarding microglial cells, it is somehow surprising that only a reduced number of reactive ameboid microglial cells express Cu/Zn SOD, and that this occurs very transiently, as in some circumstances activated microglia produce large amounts of oxygen radicals after a lesion including O_2_^-. ^[66].

We believe that the results presented here highlight the importance of *in vivo *cell-localization studies for antioxidants, as some of the reactive species like O_2_^-. ^do not diffuse across cell membranes and thus will most probably react within the cell where it is formed.

## Conclusion

In conclusion, we show that in the intact immature rat brain during the plasticity window, Cu/Zn SOD is mainly expressed in neurons, though it is also expressed at the central nervous system boundaries like the glia limitans or the ependymal cells. Moreover, we show that brain Cu/Zn SOD expression levels vary after an excitotoxic injury: it is rapidly downregulated in neurons, rendering the affected neurons even more susceptible to oxidative damage, and later on it is upregulated in highly hypertrophic astrocytes. Therefore, as no changes in cell specificity are found in the immature versus the adult brain, further studies would be needed to elucidate the mechanisms underlying the different susceptibility to oxidative damage in these two lesion paradigms.

## List of abbreviations

SOD: superoxide dismutase; O_2_^-.^: superoxide; H_2_O_2_: hydrogen peroxide; NO^.^: nitric oxide; COX: cycloxygenase; CNS: central nervous system; NFκB: nuclear factor kappa B; NMDA: N-methyl-D-aspartate; TUNEL: terminal dUTP nick end labelling; DAPI: 4, 6-diamino-2-phenylindole; GFAP: glial fibrilary acidic protein.

## Competing interests

The author(s) declare that they have no competing interests.

## Authors' contributions

HP carried out part of the brain lesions and animal work, performed most of the immunohistochemical and western blot studies, conceived the study and drafted the manuscript. LA carried out part of the brain lesions and animal work, participated in the design of the study and helped to draft the manuscript. MF carried out part of the immunohistochemistry and helped to draft the manuscript. BC and BG coordinated and supervised the development of the study, were responsible for the project giving economical support and helped in the last version of the manuscript. All authors read and approved the final manuscript.
